# Decreased levels of circulating cytokines VEGF, TNF-β and IL-15 indicate PD-L1 overexpression in tumours of primary breast cancer patients

**DOI:** 10.1038/s41598-020-80351-9

**Published:** 2021-01-14

**Authors:** Zuzana Cierna, Bozena Smolkova, Dana Cholujova, Paulina Gronesova, Svetlana Miklikova, Marina Cihova, Jana Plava, Michal Mego

**Affiliations:** 1 Department of Pathology, Faculty of Medicine, Comenius University, Sasinkova 4, 811 08 Bratislava, Slovakia; 2 Department of Pathology, Faculty Hospital, A. Zarnova 11, 917 75 Trnava, Slovakia; 3 Cancer Research Institute, Biomedical Research Center of the Slovak Academy of Sciences, Dubravska Cesta 9, 845 05 Bratislava, Slovakia; 4 2nd Department of Oncology, Faculty of Medicine, Comenius University and National Cancer Institute, Klenova 1, 833 10 Bratislava, Slovakia; 5 Translational Research Unit, Faculty of Medicine, Comenius University, Klenova 1, 833 10 Bratislava, Slovakia

**Keywords:** Prognostic markers, Breast cancer

## Abstract

Programmed death ligand 1 (PD-L1) overexpression has been associated with poor clinical outcomes in several human cancers whose increased malignant behaviour might be related to PD-L1 mediated systemic immunological tolerance. This study aims to verify if circulating cytokines may serve as a proxy for non-invasive identification of sensitive prognostic biomarkers reflecting tumour and its microenvironment. Immunohistochemistry was used to measure PD-L1 expression in tumour tissue sections of 148 chemonaïve breast cancer (BC) patients. The panel of 51 cytokines was analysed using multiplex bead arrays. High PD-L1 expression in tumours was associated with shorter progression-free survival (HR 3.25; 95% CI 1.39–7.61; P = 0.006) and low circulating levels of three multifunctional molecules; VEGF, TNF-β and IL-15 (P = 0.001). In multivariate analysis, patients with low VEGF had 4.6-fold increased risk of PD-L1 overexpression (P = 0.008), present in 76.5% of patients with all these three cytokines below the median (vs. 35.6% among the others; P = 0.002). The area under the curve value of 0.722 (95% CI 0.59–0.85; P = 0.004) shows that this combination of cytokines has a moderate ability to discriminate between PD-L1 high vs. PD-L1 low patients. Plasma cytokines, therefore, could serve as potential non-invasive biomarkers for the identification of high-risk BC cases.

## Introduction

Breast cancer (BC) is one of the most commonly diagnosed cancer types among women. Current treatment methods involving surgery, chemotherapy, radiation or targeted therapies have made considerable progress, especially when the disease is diagnosed at an early stage^1^. However, BC has been long regarded as difficult to treat with immunotherapy, because it is considered immunologically “cold”. Immunologically cold tumours are cancers, which are not recognized or haven’t provoked a strong immune system response in contrast to so-called hot tumours. By secreting immunosuppressive cytokines, chemokines and growth factors they turn down the normal immune response and the movement of T cells into the tumour^2^. Therefore, the understanding of the patient’s immune system derangement in BC has been the focal point of attention in recent decades^3^. Previous studies have demonstrated that the tumour can directly inhibit the immune cells’ function by affecting their responses, down-regulating the cellular receptors or by suppressing their mechanisms of action^4^. The programmed death-1 (PD-1) ligand 1 (PD-L1) also known as cluster of differentiation 274 (CD274) is a protein which has been speculated to play a major role in suppressing the adaptive immune system response. To evade anti-tumour immunity, several human cancer cells express high levels of PD-L1, and its blockade reduces tumour growth in the presence of immune cells^5^. PD-L1 expression level is used to select patients for anti-PD-1/L1 antibody therapy with the overall different success rate for patients with constitutive PD-L1 expression, which is a result of a genetic event, in contrast to those with inducible PD-L1 positivity/negativity in response to T cell infiltrates^6^. The recent demonstration of the single-agent activity of PD-L1 and PD-1 antibodies in BC patients generated hope that BC can also be made amenable to immunotherapy^7^. However, the expression of PD-L1 in breast tumour cells and associated stromal cells has been shown to be modest and variable^8,9^. Moreover, PD-L1 expression in relation to prognosis remains controversial, and associations show better, worse or no effect^7,10^. Therefore, further investigation of PD-L1 in BC and its effect on prognosis is required to increase understanding of the biologic processes governing PD-L1 expression and its interaction with other factors in the tumour immune microenvironment.

Based on the hypothesis that increased malignant behaviour (higher tumour grade, positive nodal status, CTC dissemination) might be associated with PD-L1 mediated systemic immune tolerance, we measured in this study expression of 51 cytokines in the peripheral blood of BC patients with the aim to identify non-invasive, PD-L1-mediated surrogate markers of immune suppression.

## Methods

### Study population

The present case–control study, including 148 chemonaïve invasive primary BC patients with stages I–III treated by surgery from March 2012 to February 2015, was nested within the larger translational study (Protocol TRU-SK 002; Chair: M. Mego). Selection of participants was based on the availability of cytokine measurement data. Mean age of included patients was 59.7 (range 35.4–83.1) years. Formalin-fixed paraffin-embedded (FFPE) tumour tissue and peripheral blood were collected from each participant. Patients suffering from a concurrent malignancy other than non-melanoma skin cancer in the previous 5 years were excluded. Relevant clinicopathologic data were recorded for each case. Briefly, 120 (81.1%) patients were older than 50 years, 43 (29.1%) were diagnosed with T-stage II or III, 54 (36.7%) had lymph node positivity (N+) and 37 (25%) presented with lymphovascular invasion (LVI). Histological subtypes consisted of 127 (85.8%) invasive ductal carcinomas (IDCs) and 21 (14.2%) invasive lobular, tubular or mucinous carcinomas. Histological grade 3 (high grade) was diagnosed in 56 (38.4%), hormone receptor (HR) negativity in 13 (8.8%), HER2 positivity in 21 (14.2%) and high Ki-67 proliferation (cut-off 14%) in 62 (41.9%) patients. The Institutional Review Board of the National Cancer Institute of Slovakia approved this study and written informed consent was obtained from all participants before study enrolment. All methods and experiments were carried out in accordance with relevant guidelines and regulations.

### Cytokine assessment

Plasma from 1 ml of ethylenediaminetetraacetic acid (EDTA)-anticoagulated peripheral blood was used for the analysis of 51 cytokines. After 10-min centrifugation at 5000 rpm, the supernatants were filtered through sterile 0.22 µm filters. Plasma aliquots were stored at − 80 °C for further analysis.

Human Group I and II cytokine and TGF beta panels were analysed using multiplex bead arrays (Bio-Plex 200 system, Bio-Rad Laboratories, Hercules, CA, USA). Human Group I 27-plex panel included following targets: IL-1beta, IL-1r alpha, IL-2, IL-4, IL-5, IL-6, IL-7, IL-8, IL-9, IL-10, IL-12 (p70), IL-13, IL-15, IL-17, Basic FGF, Eotaxin, G-CSF, GM-CSF, IFN-gamma, IP-10, MCP-1 (MCAF), MIP-1alpha, MIP-1beta, PDGF-BB, RANTES, TNF-alpha and VEGF. Group II 21-plex panel contained targets: IL-1alpha, IL-2Ralpha, IL-3, IL-12 (p40), IL-16, IL-18, CTACK, GRO-alpha, HGF, IFN-alpha2, LIF, MCP-3, M-CSF, MIF, MIG, beta-NGF, SCF, SCGF-beta, SDF-1alpha, TNF-beta (TNF-β) and TRAIL. TGF-beta 1, TGF-beta 2, TGF-beta 3 were analysed using Bio-Plex Pro TGF-beta 3-plex immunoassay following manufacturer’s instructions. Samples and premixed cytokine standards were diluted and incubated with colour-coded magnetic beads conjugated with monoclonal antibodies in a 96-well filter plate for 30 min (2 h for TGF-beta assay). Samples were activated with 1 N HCl for 10 min, then neutralized with 1.2 N NaOH/0.5 M HEPES (Applichem, Darmstadt, Germany) and assayed immediately after neutralization step. After incubation with biotinylated detection antibody, each captured analyte was detected by the addition of streptavidin–phycoerythrin and quantified by a BioPlex suspension array reader (Bio-Rad Laboratories). Cytokine concentrations (pg/ml) were calculated with Bio-Plex Manager 4.0 software using 5-parameter logistic curve fitting as published previously^11^.

### Tissue microarray construction

According to tumour histology, one or two representative tumour areas were identified on the hematoxylin and eosin sections. Sections were matched to their corresponding wax donor blocks, and 3-mm diameter cores of the tumour were removed from the donor blocks with the multipurpose sampling tool Harris Uni-Core (Sigma-Aldrich, Steinheim, Germany) and inserted into the recipient master block. The recipient block was cut into 5-μm sections, which were transferred to coated slides.

### Immunohistochemical staining

The slides were de-paraffinized and rehydrated in phosphate-buffered saline solution (10 mM, pH 7.2). The tissue epitopes were de-masked using the automated water bath heating process in Dako PT Link (Dako, Glostrup, Denmark) and the slides were incubated in TRIS–EDTA retrieval solution (10 mM TRIS, 1 mM EDTA pH 9.0) at 98 °C for 20 min. The slides were subsequently incubated for 1 h at room temperature with the primary rabbit monoclonal antibody against PD-L1 (Abcam [EPR1161(2)]: AB174838) diluted 1:200 in Dako REAL antibody diluent (Dako, Glostrup, Denmark) and immunostained using anti-mouse/anti-rabbit immuno-peroxidase polymer (EnVision FLEX/HRP, Dako, Glostrup, Denmark) for 30 min at room temperature. For visualization, the slides reacted with diaminobenzidine substrate-chromogen solution (DAB, Dako, Glostrup, Denmark) for 5 min. Finally, the slides were counterstained with hematoxylin. PD-L1 positivity of lymphocytes in the tonsil was used as a positive control, same tissue with omitting the primary antibody served as a negative control. The percentage of positive cells was estimated on a scale of 0–100%. Staining intensity was scored on the scale from 0 to 3 (0-no staining, 1-weak, 2-moderate, and 3-strong staining). Weighted histoscore was then calculated by multiplying the percentage and intensity scores, yielding values from 0–300. PD-L1 expressions were graded as low (0) or high (1–300).

### Statistical analysis

The normality of distribution for continuous variables was assessed by the Kolmogorov–Smirnov or Shapiro–Wilk tests. Continuous data were summarized as arithmetic means with standard deviations (SDs) or medians with ranges according to data distribution. Two group comparisons were tested using Student's t-test or Mann–Whitney *U*-test. As cytokines were non-normally distributed, the Dunn or Dunn–Bonferroni post hoc method was applied following a significant Kruskal–Wallis test when more than two groups were compared. Correlations were analysed using the Spearman rank correlation test. The values of cytokines in patient plasma were dichotomized with the cut-off level of median into two categories: low (values below median) or high (values above the median). Categorical variables are presented as frequencies and percentages. Immune parameters associated with clinical features were determined by bivariate analysis. Pearson chi-square or Fisher exact tests were used to examine the association between tumour characteristics and dichotomized cytokine plasma levels. The logistic regression was applied to identify variables associated with PD-L1 expression in tumours. This determination included computation of the risk estimate presented as estimated odds ratio (OR) and 95% confidence interval (CI) for the OR. Each model includes age, significant clinicopathologic characteristics (Table 1) and cytokines significant in univariate analysis. A backward model selection was conducted, and the final fitted model is presented. The mean follow-up period was calculated as a mean observation time among all patients and among those still alive at the time of their last follow-up. Progression-free survival (PFS) was calculated as the interval from the date of sampling (mostly date of surgery) to the date of progression, death or last adequate follow-up. PFS curves for PD-L1 and each cytokine were estimated by the Kaplan–Meier method and the differences between groups were compared with log-rank or Breslow tests. Statistically significant and borderline variables (*P* values ≤ 0.1) were included in the Cox proportional hazard model, applied to estimate the hazard ratio of each covariate and to adjust for potential confounders. Statistical significance was determined as *P* < 0.05 with a two-sided test. All data were analysed using the SPSS software package version 23 (SPSS, Inc., Chicago, IL, US).

**Table 1:** PD-L1 expression in relation to clinical characteristics of patients.

Variables	Categories	n (%)	PD-L1 in tumour n (%)			PD-L1 in stroma n (%)		
			Low	High	P	Low	High	P
Age (years)	≤ 50	28 (18.9)	11 (50.0)	11 (50.0)	0.418	16 (76.2)	5 (23.8)	**0.004**
	> 50	120 (81.1)	60 (59.4)	41 (40.6)		95 (95.0)	5 (5.0)	
T-stage	T1	105 (70.9)	54 (62.8)	32 (37.2)	0.083	80 (94.1)	5 (5.9)	0.144
	T2 and more	43 (29.1)	17 (45.9)	20 (54.1)		31 (86.1)	5 (13.9)	
Histology	IDC	127 (85.8)	56 (53.3)	49 (46.7)	**0.017**	93 (90.3)	10 (9.7)	0.168
	Others	21 (14.2)	15 (83.3)	3 (16.7)		18 (100.0)	0 (0.00)	
Grade	Low and intermediate	90 (61.6)	52 (69.3)	23 (30.7)	**0.001**	70 (95.9)	3 (4.1)	**0.033**
	High	56 (38.4)	18 (39.1)	28 (60.9)		39 (84.8)	7 (15.2)	
N-stage	N0	93 (63.3)	45 (57.7)	33 (42.3)	0.880	70 (90.9)	7 (9.1)	0.688
	N+	54 (36.7)	26 (59.1)	18 (40.9)		40 (93.0)	3 (7.0)	
M-stage	M0	148 (100)	71 (58.2)	51 (41.8)	NA	110 (91.7)	10 (8.3)	NA
	M+	0	0	0		0	0	
LVI	Absent	111 (75.0)	57 (60.6)	37 (39.4)	0.239	89 (96.7)	3 (3.3)	**0.000**
	Present	37 (25.0)	14 (48.3)	15 (51.7)		22 (75.9)	7 (24.1)	
HR status^a^	Negative	13 (8.8)	3 (25.0)	9 (75.0)	**0.016**	10 (83.3)	2 (16.7)	0.265
	Positive	135 (91.2)	68 (61.3)	43 (38.7)		101 (92.7)	8 (7.3)	
HER2 status	Negative	127 (85.8)	61 (57.0)	46 (43.0)	0.678	96 (91.4)	9 (8.6)	0.753
	Amplified	21 (14.2)	10 (62.5)	6 (37.5)		15 (93.8)	1 (6.3)	
p53	Negative	94 (63.9)	42 (56.0)	33 (44.0)	0.698	67 (90.5)	7 (9.5)	0.571
	Positive	53 (30.1)	28 (59.6)	19 (40.4)		43 (93.5)	3 (6.5)	
bcl2	Negative	45 (30.4)	14 (41.2)	20 (58.8)	**0.022**	28 (82.4)	6 (17.6)	**0.019**
	Positive	103 (69.6)	57 (64.0)	32 (36.0)		83 (95.4)	4 (4.6)	
Ki-67^b^	Low	86 (58.1)	47 (68.1)	22 (31.9)	**0.008**	64 (94.1)	4 (5.9)	0.281
	High	62 (41.9)	24 (44.4)	30 (55.6)		47 (88.7)	6 (11.3)	
Tumour subtypes	Luminal A	86 (58.1)	43 (60.6)	28 (39.4)	0.202	65 (92.9)	5 (7.1)	0.653
	Luminal B	30 (20.3)	15 (60.0)	10 (40.0)		22 (91.7)	2 (8.3)	
	HER2 positive	21 (14.2)	10 (62.5)	6 (37.5)		15 (93.8)	1 (6.3)	
	Triple-negative	11 (7.4)	3 (27.3)	8 (72.7)		9 (81.8)	2 (18.2)	

The number of analysed samples for individual measured variables from the total number of 148 enrolled patients is shown in the table.

Significant results are highlighted in bold.

IDC, invasive ductal carcinoma; LVI, lymphovascular invasion; HR, hormonal receptor.

^a^Negative for both or positive for either with cut-off 1%.

^b^Cut-off 14%.

## Results

### Association between PD-L1 expression in the primary tumour and patient/tumour characteristics

**Figure 1:**
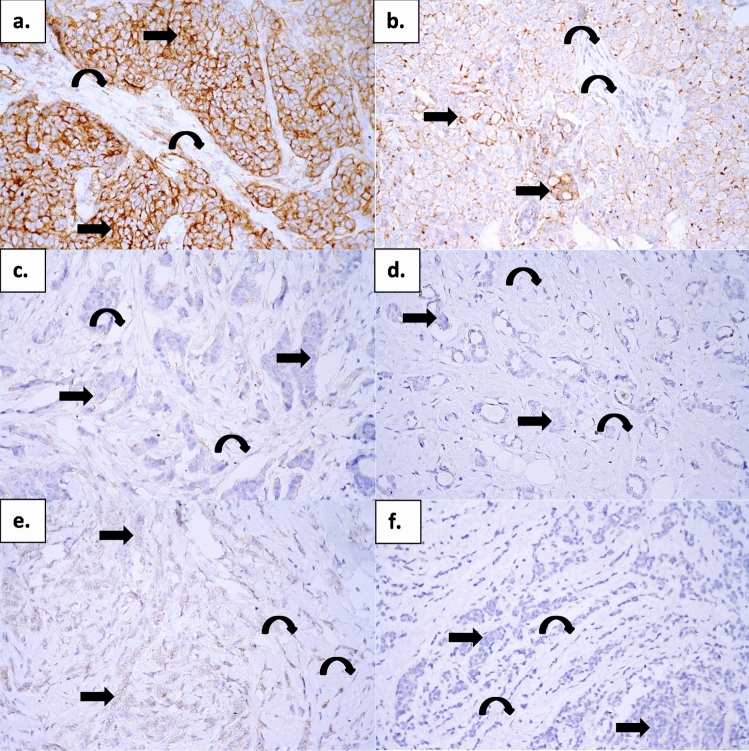
PD-L1 expression in primary breast tumours. Immunohistochemical reaction with an anti-PD-L1 monoclonal antibody. Original magnification ×400 visualisation with 3,3′-diaminobenzidine (brown colour). (**a**) (IDC grade 3) strong positivity in tumour cells (arrow) and weak positivity in stromal cells (curved arrow), (**b**) (IDC grade 3) weak to moderate positivity in tumour cells (arrow) and negativity in stromal cells (curved arrow), (**c**) (IDC grade 3) focal weak positivity in tumour cells (arrow) and negativity in stromal cells (curved arrow), (**d**) (IDC grade 1) negativity in tumour cells (arrow) and stromal cells (curved arrow), (**e**) (ILC) weak positivity in tumour cells (arrow) and negativity in stromal cells (curved arrow), (**f**) (ILC) negativity in tumour cells (arrow) and stromal cells (curved arrow). IDC, invasive ductal carcinoma; ILC, invasive lobular carcinoma.

High PD-L1 expression in tumours was associated with several adverse clinical characteristics (Table 1). It was more frequent in patients with high grade (60.9% vs. 30.7% in those with low and intermediate grades, P = 0.001), high Ki-67 proliferation (55.6% vs. 31.9% in Ki-67 low patients, P = 0.008) and in HR negative patients (75.0% vs. 38.7% in HR-positive patients, P = 0.016). Patients with IDC had high PD-L1 in tumours more frequently than patients with other subtypes (46.7% vs. 16.7%, P = 0.017) and it was also more frequent in B-cell Lymphoma 2 (bcl2) negative patients (58.8% vs. 36.0% in positive patients, P = 0.022). Elevated PD-L1 expression in the stroma was present in only 10 (8.3%) of 121 patient tissues. Despite low numbers, it was significantly associated with high grade (15.2% vs. 4.1% in patients with the low and intermediate grade, P = 0.033), presence of LVI (24.1% vs. 3.3% in LVI absence; P = 0.000) and bcl2 negativity (17.6% vs. 4.6% for bcl2 positive patients, P = 0.019). PD-L1 expression in tumour and stroma did not differ between molecular BC subtypes, although triple-negative patients had significantly higher PD-L1 expression in tumours than non-triple-negative patients (median 15%, range 0–115% vs. median 0%, range 0–120%; P = 0.032). Representative images of PD-L1 expression in primary breast tumours are shown in Fig. 1.

### Prognostic significance of PD-L1 expression in the primary tumour

**Figure 2:**
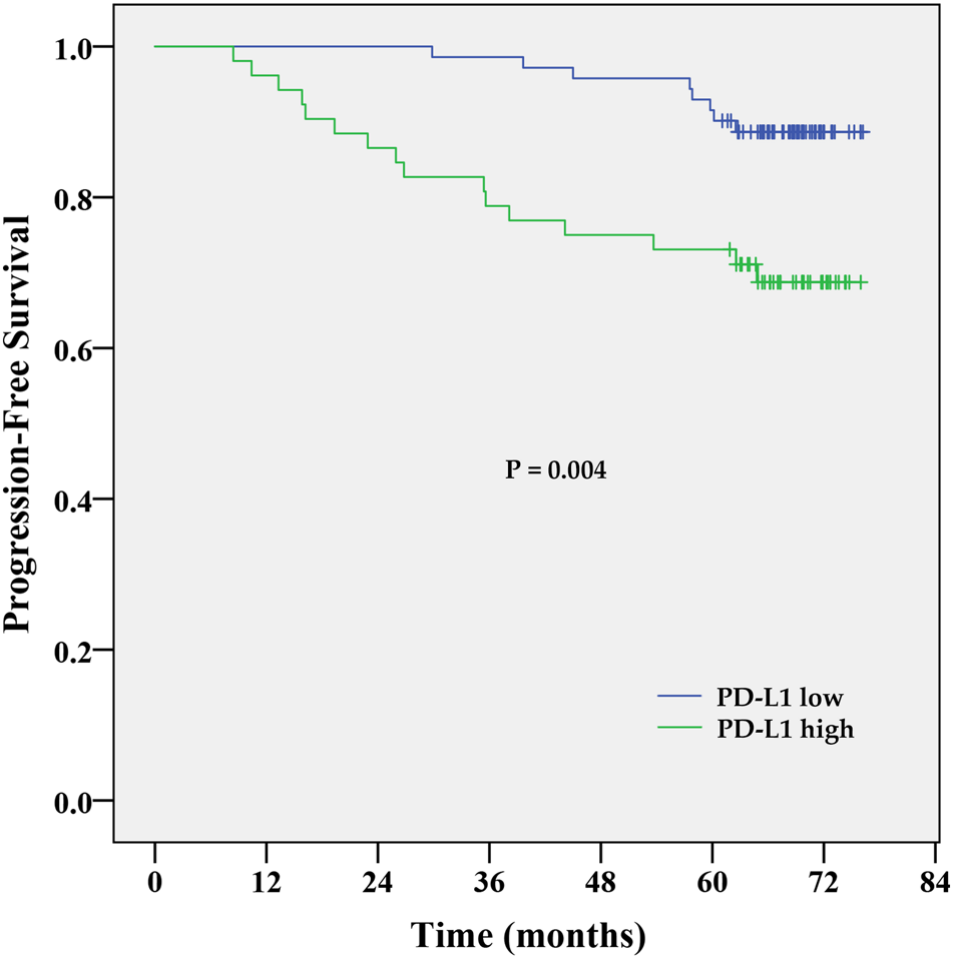
Kaplan–Meier PFS estimates for the PD-L1 expression in the tumour. Patients with high PD-L1 expression had shorter PFS compared to patients with low PD-L1 expression, P = 0.004 by log-rank test.

**Figure 3:**
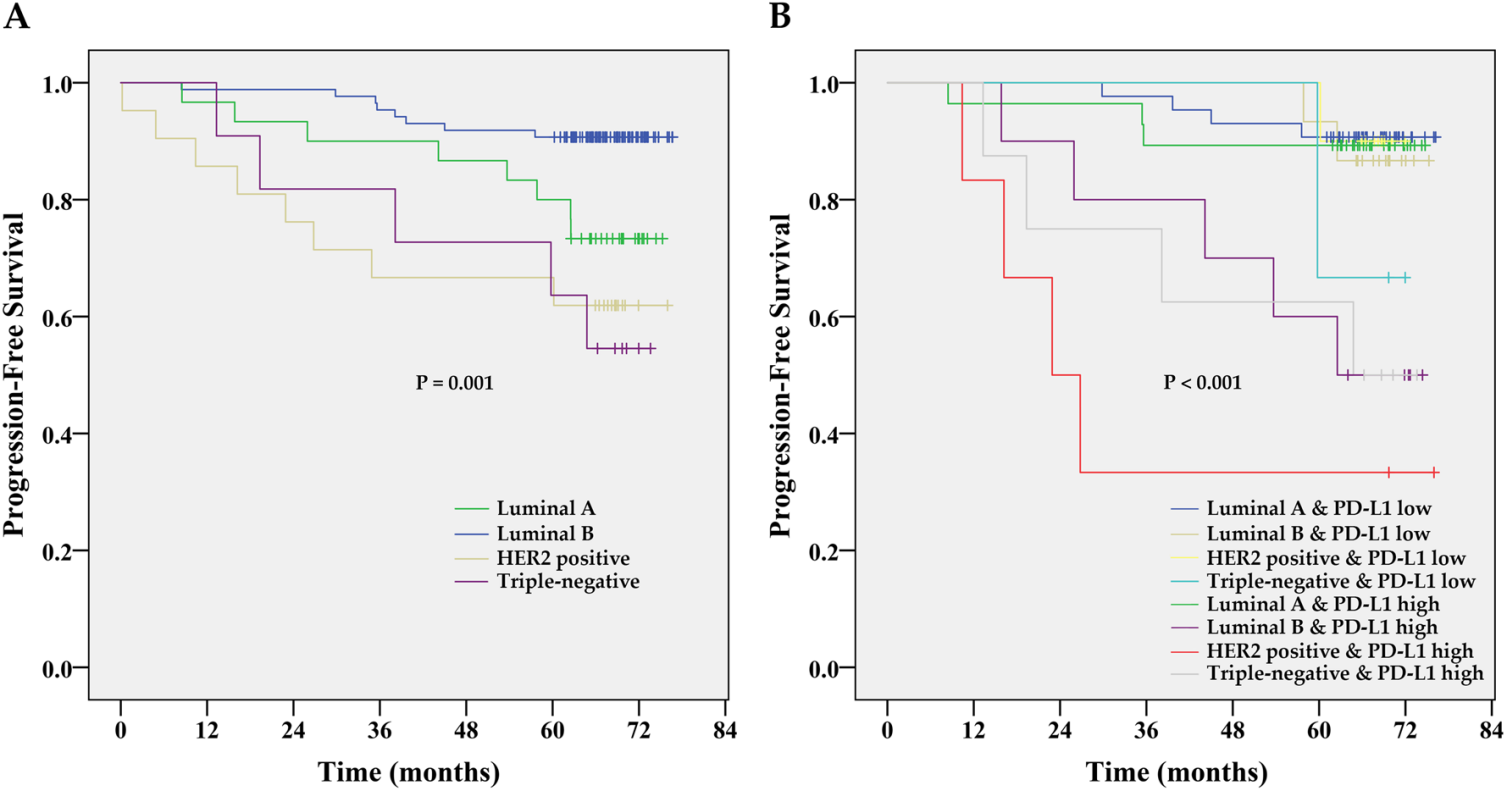
Kaplan–Meier PFS estimates for individual molecular subtypes (**A**, P = 0.001 by log-rank test) and molecular subtype-stratified PD-L1 expression in the tumour (**B**, P < 0.001 by log-rank test).

At a median follow-up time of 67.1 months (range 0.2 to 76.7 months), 29 patients (19.6%) had experienced a PFS event, and 16 patients (11.5%) had died. Due to the immaturity of overall survival data, we present the PFS analysis only. The Kaplan–Meier PFS estimates for PD-L1 expression in tumours are shown in Fig. 2. While estimated 2-year PFS rates in patients with low PD-L1 expression were 89%, it was 69% only for those with high PD-L1 expression. The 5-year PFS rates were identical to 2-year estimates. High PD-L1 expression was associated with decreased PFS (HR 3.253; 95% CI 1.39–7.61; P = 0.006) by univariate Cox proportional hazard regression analysis. After stratification for clinical subtypes (Fig. 3), high PD-L1 expression indicates shorter PFS (log-rank test), especially in HER2 positive (P = 0.008) and luminal B patients (P = 0.033). A significant difference was not found between PD-L1 high and PD-L1 low patients with luminal A subtype (P = 0.810), where both groups had a good prognosis. Triple-negative patients (P = 0.584) had poor prognosis irrespective of their PD-L1 status. The fact that PD-L1 high expression was identified in 75% of triple-negative patients, which is almost double the incidence than in other clinical subtypes (Table 1), may explain the negative result of the statistical analysis for this subgroup. Multivariate analysis (Table 2) confirmed high PD-L1 expression as an independent prognostic factor for PFS (HR = 3.361; 95% CI = 1.39–8.13; P = 0.007). High PD-L1 expression increased the risk of poor outcome more than 3 times. However, we did not find a significant association between the PD-L1expression in stroma and PFS.

**Table 2:** Cox proportional hazard regression analysis for the association between PD-L1 expression in tumour, molecular subtypes and PFS adjusted for age.

	HR	95% CI	P
High PD-L1 expression	3.361	1.39–8.13	0.007
**Molecular subtypes**			0.025
Luminal B	3.152	1.10–9.00	0.032
HER positive	4.677	1.46–14.97	0.009
Triple-negative	4.045	1.27–12.92	0.018

### Association between PD-L1 and plasma cytokines

**Figure 4:**
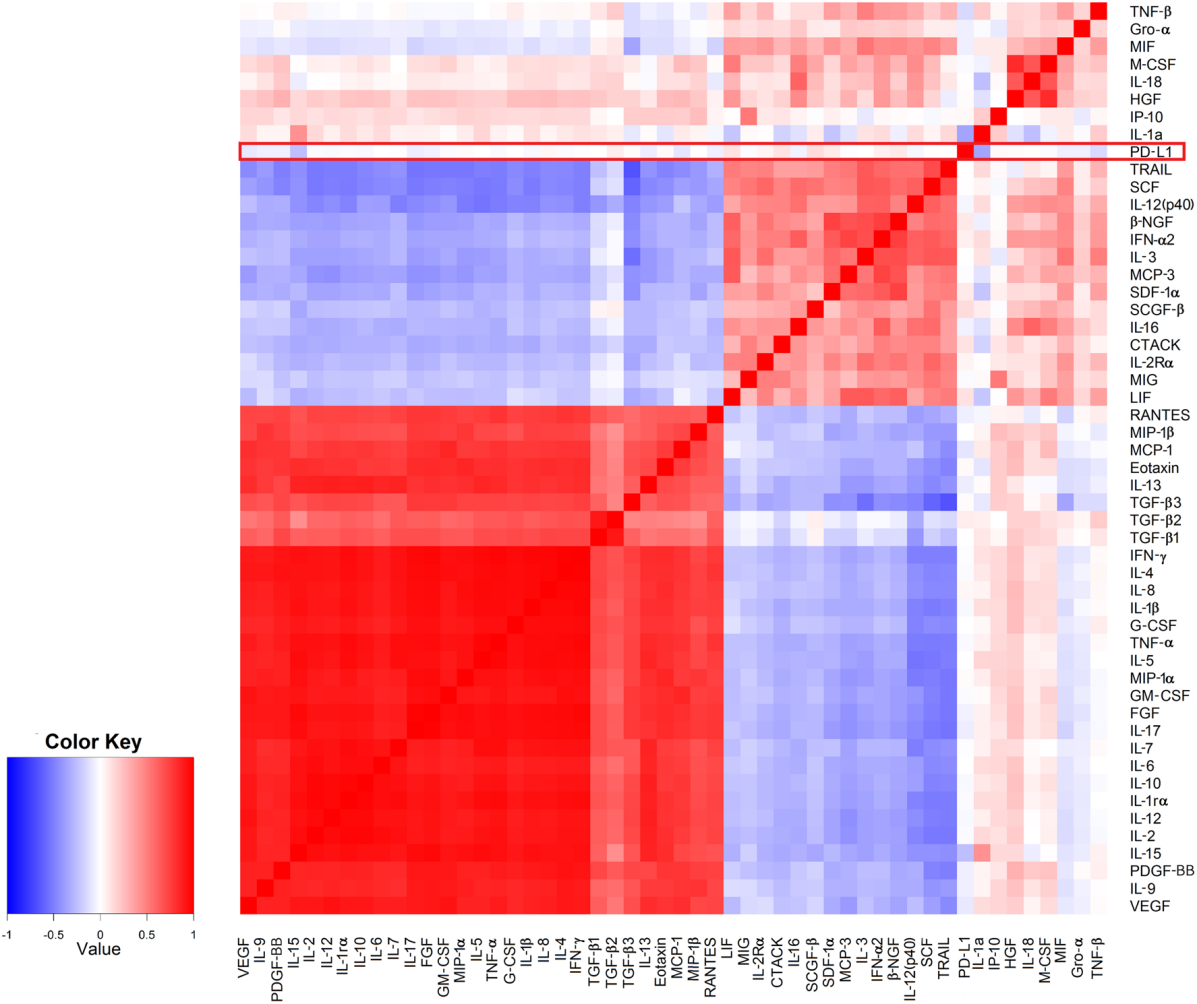
The correlations between PD-L1 and individual cytokines.

**Figure 5:**
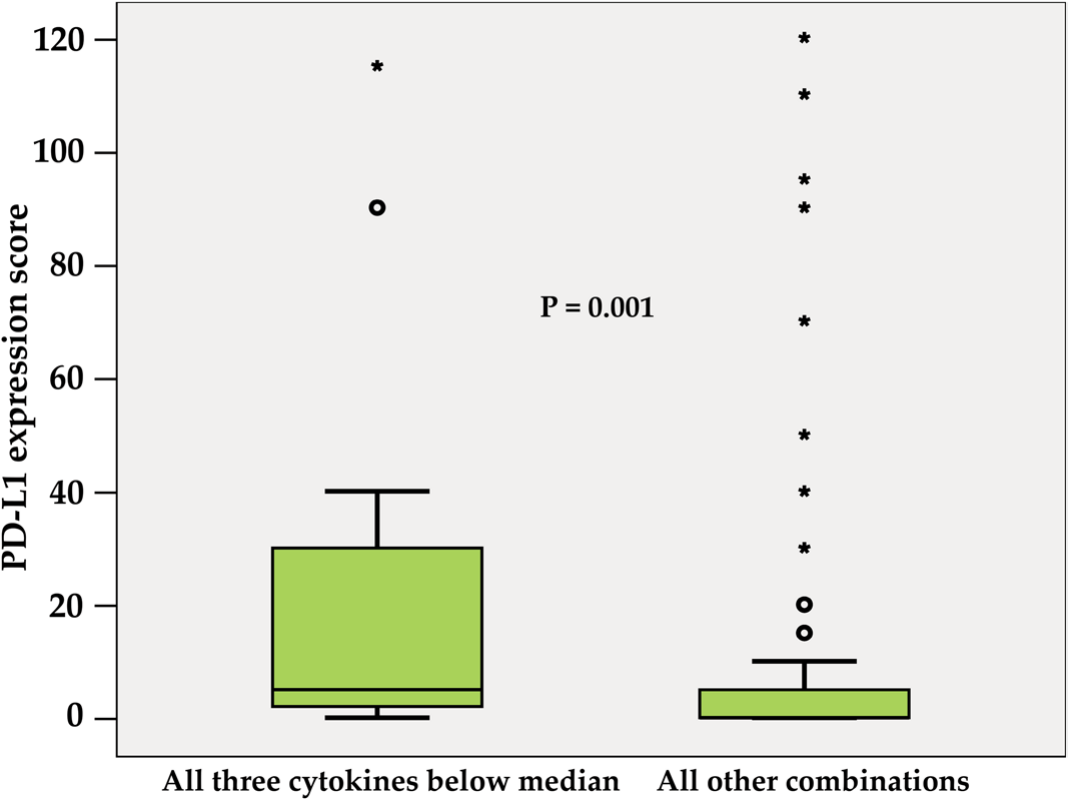
PD-L1 expression in tumours of BC patients stratified by combined VEGF, TNF-β and IL-15 levels (all three below the median vs. all other combinations). The length of the boxes is the interquartile range (IQR) that represents values between the 75th and 25th percentiles. Values more than three IQRs from the end of a box are labelled as extreme (*). Values more than 1.5 IQRs but less than 3 IQRs from the end of the box are labelled as outliers (O). The median is depicted by a horizontal line.

**Figure 6:**
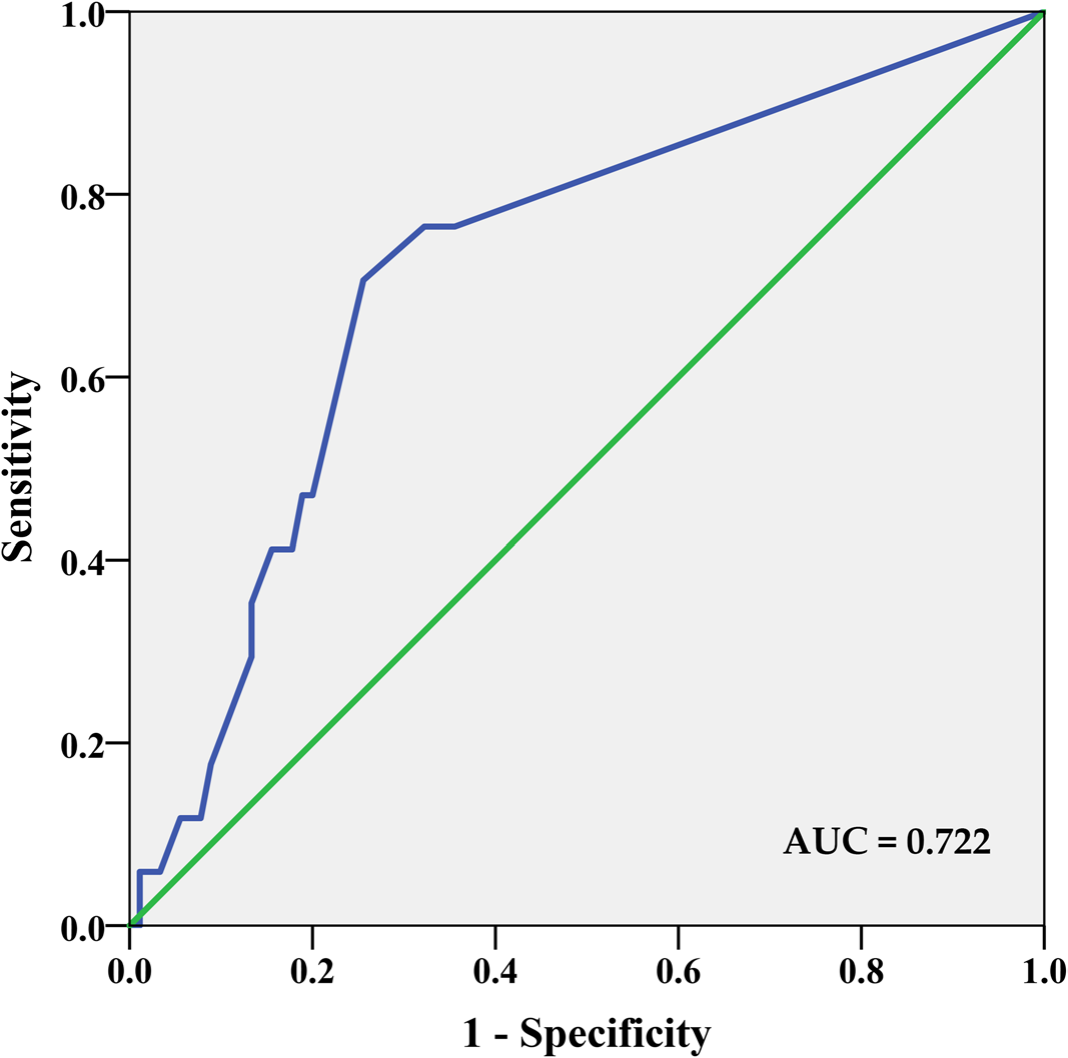
The area under a curve (AUC) of receiver operating characteristic (ROC) analysis for PD-L1 expression in tumour based on IHC data. ROC analysis revealed that low circulating VEGF, IL-15, and TNF-β levels (all three below the median vs. all other combinations) has significant sensitivity and specificity to discriminate between patients with low and high PD-L1 expression in tumours. The area under the curve (AUC) value of 0.722 (95% CI 0.59–0.85; P = 0.004).

The correlations between PD-L1 and individual cytokines are depicted in Fig. 4. Among the 51 analysed plasma cytokines, the only significant correlation was detected with interleukin 15 (IL-15) (r = − 0.249; P = 0.013). However, as shown in Table 3, high PD-L1 was identified more frequently in patients with circulating values of vascular endothelial growth factor (VEGF), tumour necrosis factor-beta (TNF-β) and interleukin 15 (IL-15) below median (54.7% vs. 33.9%, P = 0.025 for VEGF, median value = 49.68 pg/ml; 52.4% vs. 32.2%, P = 0.042 for TNF-β, median value = 0.37 pg/ml and 58.3% vs. 38.0%, P = 0.044 for IL-15, median value = 17.31 pg/ml). Patients with all three cytokines below the median (Fig. 5) had significantly higher mean PD-L1 score value (P = 0.001). In this group, high PD-L1 expression was found in 76.5%, while it was present only in 35.6% of patients with at least one high-expression cytokine (P = 0.002). Multivariate analysis confirmed the association between VEGF and PD-L1 expression. Low VEGF values were associated with a 4.6-fold higher risk of having high PD-L1 (P = 0.008) (Table 4). The model was able to correctly classify 75.6% of PD-L1 low and 83.8% of PD-L1 high patients, with an overall success rate of 79.5%. Receiver operating characteristic (ROC) curve analysis was applied to determine the diagnostic ability of all three plasma cytokines combined to correctly classify subjects according to their PD-L1 expression in tumours. The area under curve (AUC) value 0.722 (95% CI 0.590–0.853; p = 0.004) on corresponding ROC curve (Fig. 6) shows that model performed fairly well regarding its predictive ability to discriminate between PD-L1 high vs. PD-L1 low subjects.

**Table 3:** Cytokines significantly associated with PD-L1 in univariate analysis.

	Cut-off values (pg/ml)	PD-L1 low	PD-L1 high	P
VEGF	< 49.68	24 (45.3)	29 (54.7)	0.025
	≥ 49.68	41 (66.1)	21 (33.9)	
TNF-β	< 0.37	20 (47.6)	22 (52.4)	0.042
	≥ 0.37	40 (67.8)	19 (32.2)	
IL-15	< 17.31	20 (41.7)	28 (58.3)	0.044
	≥ 17.31	31 (62.0)	19 (38.0)	
Combination	All three below median	4 (23.5)	13 (76.5)	0.002
	All other combinations	58 (64.4)	32 (35.6)	

The number of analysed samples for individual cytokines from the total number of 148 enrolled patients is shown in the table.

**Table 4:** Binary logistic regression (adjusted for age) for the relationship between analysed cytokines and clinicopathological characteristics with PD-L1.

Variable	OR	95% CI	Sig
IDC	4.26	0.77–23.71	0.098
High grade	4.92	1.54–15.67	**0.007**
bcl2 negative	3.61	0.97–13.38	0.055
VEGF < 49.68 pg/ml	4.60	1.48–14.32	**0.008**

Variables entered in step 1: age, histology, grade, bcl2, HR status, ki-67 proliferation, VEGF, TNF-β, IL-15;  − 2 Log likelihood = 77.83; R^2^ (Cox and Snell) = 0.32; R^2^ (Nagelkerke) = 0.43.

Significant results are highlighted in bold.

IDC, invasive ductal carcinoma.

## Discussion

Although analysis of blood-based biomarkers has remarkable advantages compared to traditional methods of cancer management, liquid biopsy in BC remains a complementary approach to classical tissue biopsies so far. New blood-based biomarkers can therefore help to improve early diagnosis and screening, refine prognosis, allow long term monitoring of disease progression and guide clinical decisions. All these advantages make them particularly attractive in immunotherapy, which in BC has not yet been successful, with response rates in patients with locally advanced or metastatic disease up to 10%^12–14^. However, recent evidence of significant immune infiltration in triple-negative BC and the success of IMpassion130 trial prove the potential benefit of immunotherapy in BC^15^. The better understanding of the complex relationships between the host, tumour and its microenvironment will allow for more efficient patient stratification and tailored management.

Tumour formation relies on oncogenic changes within tumour cells and their interaction with the stromal environment. During this process, tumour cells can lose their immunogenic tumour antigens and acquire immunosuppressive properties, which allow them to evade immune-defence system^16^. Although PD-1 and its ligand PD-L1 have been identified as negative immunoregulatory molecules and their enhanced expression has been found to correlate with a poor prognosis for several cancer types, valid conclusion for other types cannot be currently drawn^17^. Despite many controversies regarding the prognostic and clinicopathological value of PD-L1 protein expression in BC^18–20^, our data, in concordance with others, support this inverse association^7,18^. The largest systematic review so far^7^ summarized the results of thirty-seven articles, defining the distribution of PD-L1 expression in cancer subtypes and its association with patient outcome. The authors showed that PD-L1 varied greatly across subtypes (0–83%) and further investigation is required^7^. The recent meta-analysis of Huang et al.^19^ associated PD-L1 expression with shorter disease-free survival and overall survival, but Stovgaard et al. also showed studies with the opposite findings or with the effect detected only in specific BC subtype^7^. Similar to our results, Li et al.^20^ showed PD-L1 high expression to be significantly associated with high tumour grade, negative hormone receptor status and high Ki-67. Among the main reasons for conflicting results, different cut-off values and scoring systems, various antibodies used for IHC detection, intracellular oncogenic variations, intra-tumoural heterogeneity and dynamic alteration in PD-L1 expression have been discussed.

In this context, the predictive value of PD-L1 expression in BC remains vague and deserves further investigation^7^. Nevertheless, an increasing number of studies has shown that PD-1/PD-L1 signal blockade can reverse immunosuppression and might serve as a promising clinical strategy for a precisely stratified subgroup of patients^9,21,22^. Predictive biomarkers enabling identification of patients who will most likely respond to immunotherapy will allow decreasing current unsatisfactory low response rate^23^. Recently, soluble forms of PD-1 and PD-L1 have been detected in the blood of cancer patients^24^. Based on the published data, soluble PD-L1 may facilitate the prediction of overall survival and treatment response in a specific therapy regimen or the efficacy of neoadjuvant therapy in the specific BC subtypes^25,26^. However, the data providing new clues on their potential as a diagnostic, therapeutic, or prognostic biomarker are added up gradually and especially in BC have not been fully elucidated yet.

Recently Jabeen and colleagues have shown that factors secreted by tumour, stromal or immune cells may affect the composition of patient’s serum^27^. On the contrary, serum cytokines exerting a variety of biological effects with an important regulatory role in cell growth, survival and differentiation may contribute to the progression of the malignancy. Circulating cytokine levels may, therefore, represent an interesting non-invasive biomarker of tumour-induced immune response with important predictive and prognostic value^27,28^. Nevertheless, the interplay between the tumour and the immune system is complex and difficult to decipher.

In the present work, we aimed to assess circulating cytokine levels as a surrogate marker of PD-L1-mediated immune suppression. Although we found specific correlation clusters between individual cytokines, indicating a common role for these cytokines in regulating inflammatory and immune responses, only one significant correlation between PD-L1 expression and plasmatic cytokine levels was identified. Being the main component of tumour and host crosstalk, cytokine profiles may vary depending on BC stages, patient's clinical conditions or presence of infiltrating immune cells^29,30^. Moreover, tumour plasticity (known as an epithelial-mesenchymal transition) was shown to contribute to the development of an inflammatory and immunosuppressive tumour microenvironment^31^.

We identified an inverse association between PD-L1 expression in tumour cells and the plasmatic level of VEGF and two T-helper 1 (TH1) cellular immunity-related cytokines, namely IL-15 and TNF-β. IL-15, a pleiotropic cytokine, is constitutively expressed by a large number of cell types and tissues and plays an important role in innate and adaptive immunity. IL-15 primarily stimulates proliferation and cytotoxic functions of NK and CD8 T cells leading to enhanced anti-tumour responses^32,33^. The inability of cytotoxic T-lymphocytes to eliminate tumours that lack the expression of IL-15 and stress signals proposes a crucial role of IL-15 in immune responses^34^. However, more systematic analysis of IL-15 expression in solid tumours is necessary as an opposite effect of IL-15 expression in different types of cancer has been also published. IL-15 has been shown to promote proliferation, survival, migration, invasion, and metastasis of tumour cells^35^ and directly induces the expression of PD-1 and PD-L1 in purified T cells in vitro^36^. While *IL15* deletion in tumour cells correlated with decreased IL-15 expression and poor clinical prognosis in colorectal cancers^37^, high level of IL-15 was associated with increased inflammation and poor clinical outcome in head and neck cancer^38^. In agreement with our findings, Cohen and colleagues who used immunoassay method for the quantitation of circulating inflammatory mediators, identified zero levels of IL-15 in invasive BC patients while its level was higher in ductal carcinoma in situ (DCIS). On the other hand, IL-15 amount was doubled in metastatic BC^39^. Based on these findings, we hypothesized that increased PD-L1 expression detected in BC patients with low circulating IL-15 level is a consequence of attenuated anti-tumour response proposed by Jabrie and Abadie^34^.

Furthermore, it has been suggested that IL-15 may stimulate the production of pro-angiogenic factors such as VEGF^40^. Although higher plasmatic VEGF levels are usually associated with advanced tumour stages^41^, our study shows an inverse association of low plasmatic VEGF concentrations with high PD-L1 expression in tumours. Inverse correlation between the expression of PD-L1 protein and VEGF-related genes was also observed by Joseph and colleagues in primary clear renal cell carcinoma. The authors identified the correlation of VEGF^low^ PDL-1^high^ status with an immune evasive phenotype in contrast to an angiogenic phenotype VEGF^high^ PDL-1^low^, suggesting immune suppressive effects of VEGF signalling^42^. Inverse correlation between PD-L1 expression and angiogenic factors was demonstrated also in gastro-entero-pancreatic neuroendocrine tumours^43^. Roberti and colleagues have shown potential use of VEGF as a prognostic biomarker for triple-negative BC^44^. However, the data regarding the relationship between PD-L1 and VEGF are incoherent and scanty. Moreover, the regulation of angiogenesis by the immune system with both pro- and anti-angiogenic activities was shown^40^. Tumour angiogenesis and tumour immunity share a complex relationship that deserves consideration to decipher limited antiangiogenic therapy responses and commonly occurring resistance mechanisms.

TNF was recognized as a key cytokine linking inflammation and cancer^45^. It was originally discovered as a serum protein with necrotizing effect on certain tumours in vitro. One of the initial hypothesis implicated TNF as a part of surveillance mechanisms against tumours^46^. TNF is now considered a highly pleiotropic cytokine, playing a contextual role in driving either tumour elimination or promotion^47^. The studies focusing on local effects of TNF on tumour development revealed that constitutive TNF expression at the site of malignancy exerts strong and long-term suppression of tumour growth. However, systemic administration was associated with severe toxicity^48^. Therefore, TNF-β, also called lymphotoxin-α was explored, with regard to its potential to induce an anti-tumour response, as targeted disruption of the *LTA *gene resulted in enhanced tumour growth and metastasis in vitro^49^. As a signalling molecule, TNF-β is involved in the regulation of cell survival, proliferation, differentiation and apoptosis^50^. Analysis of inflammatory mediators in BC sera by Cohen et al.^39^ has shown decreased median level of TNF-β in metastatic BC compared to DCIS. Based on these findings, we hypothesize that low TNF-β levels in the plasma of PD-L1 high patients could reflect immunosuppressive tumour signalling.

Immune escape is an important mechanism of tumour survival. It involves many factors, including immunosuppression, where PD-1/PD-L1 signalling pathway is an important player able to inhibit activation of T lymphocytes and enhance immune tolerance. This effect is mediated by a complex molecule network including growth factors, cytokines, chemokines and exosomes^51^. Their secretion enables rapid propagation of immune signalling in a multifaceted manner, characterized by a significant degree of pleiotropism, giving one cytokine the ability to act on many different cell types to mediate diverse and sometimes opposing effects^4^. Based on our findings, we can conclude that circulating cytokines may serve as a proxy for non-invasive identification of highly sensitive prognostic biomarkers reflecting tumour and its microenvironment. However, small sample size and the highly skewed distributions for cytokine expression with a subset of very high values are limitations of this study. Future research is needed to characterize additional systemic inflammatory factors and reliably identify high-risk patients as well as to find the best stimuli to change a tumour-promoting to a tumour-inhibiting state.
